# Pancreatic alpha-cell mass in the early-onset and advanced stage of a mouse model of experimental autoimmune diabetes

**DOI:** 10.1038/s41598-019-45853-1

**Published:** 2019-07-02

**Authors:** Eva Bru-Tari, Nadia Cobo-Vuilleumier, Paloma Alonso-Magdalena, Reinaldo S. Dos Santos, Laura Marroqui, Angel Nadal, Benoit R. Gauthier, Ivan Quesada

**Affiliations:** 1 Instituto de Investigación, Desarrollo e Innovación en Biotecnología Sanitaria de Elche (IDiBE), IBMC, Universidad Miguel Hernández, Elche, Spain; 2 Biomedical Research Center in Diabetes and Associated Metabolic Disorders (CIBERDEM), Madrid, Spain; 3 Department of Cell Regeneration and Advanced Therapies, Andalusian Center for Molecular Biology and Regenerative Medicine-CABIMER, Junta de Andalucia-University of Pablo de Olavide-University of Seville-CSIC, Seville, Spain

**Keywords:** Type 1 diabetes, Type 1 diabetes

## Abstract

Most studies in type 1 diabetes (T1D) have focused on the loss of the pancreatic beta-cell population. However, despite the involvement of the alpha-cell in the aetiology and complications of T1D, little is known about the regulation of the pancreatic alpha-cell mass in this disease. The need for a better understanding of this process is further emphasized by recent findings suggesting that alpha-cells may constitute a potential reservoir for beta-cell regeneration. In this study, we characterized the pancreatic alpha-cell mass and its regulatory processes in the transgenic RIP-B7.1 mice model of experimental autoimmune diabetes (EAD). Diabetic mice presented insulitis, hyperglycaemia, hypoinsulinemia and hyperglucagonemia along with lower pancreatic insulin content. While alpha-cell mass and pancreatic glucagon content were preserved at the early-onset of EAD, both parameters were reduced in the advanced phase. At both stages, alpha-cell size, proliferation and ductal neogenesis were up-regulated, whereas apoptosis was almost negligible. Interestingly, we found an increase in the proportion of glucagon-containing cells positive for insulin or the beta-cell transcription factor PDX1. Our findings suggest that pancreatic alpha-cell renewal mechanisms are boosted during the natural course of EAD, possibly as an attempt to maintain the alpha-cell population and/or to increase beta-cell regeneration via alpha-cell transdifferentiation.

## Introduction

Type 1 diabetes (T1D) is characterized by the autoimmune destruction of the pancreatic beta-cell population and the consequent decrease or lack of insulin secretion^1^. While the pancreatic beta-cell function, death and regeneration in T1D has been intensively studied^1^, the pancreatic alpha-cell changes and adaptations in this pathology remain poorly understood. Pancreatic alpha-cells have a key role in the regulation of glycaemia: glucagon secretion from these cells triggers hepatic glucose release and, ultimately, prevents hypoglycaemia^2,3^. Conversely, hyperglucagonemia is frequently found in animal models of T1D and diabetic patients, which has been related with impaired glucagon secretion and/or altered cell morphology^2–9^. In this pathological context of insulin deficit, increased plasma glucagon levels contribute to diabetic hyperglycaemia by increasing hepatic glucose output^9^. Indeed, the pancreatic alpha-cell function and mass are potential targets of therapeutic strategies in diabetes^2,3,10,11^. Several experimental and clinical studies are aimed at limiting glucagon secretion and/or action to ameliorate the condition of hyperglycaemia in diabetes by decreasing hepatic glucose output^2,11^. Additionally, it has been also proposed that pancreatic alpha-cells could be a source for beta-cell regeneration in type 1 diabetes^10^.

Despite scarcity of information, analysis of human pancreata has shown that the alpha-cell mass is commonly preserved in recent-onset T1D patients^12^, while it seems to be reduced in long-standing T1D subjects^13,14^. Although the few existing data suggest that human alpha-cells undergo a highly dynamic state during disease progression^15–17^, the regulation of the alpha-cell mass in T1D is largely unknown. Due to limitations of human samples, murine models are indispensable surrogates to study T1D pathology. Nonetheless, pending on the mouse model, different outcomes have been reported regarding the status of the alpha-cell mass and its regulatory processes involved. It has been shown that the alpha-cell mass is unchanged in the autoimmune Non-Obese Diabetic (NOD) mice^18^ as well as in mice treated with multiple low-dose of streptozotocin (STZ)^6,19^, a chemical-induced diabetic model that develops insulitis^20^. In contrast, another study reported increased alpha-cell area in this latter animal model^8^. These discrepancies might be partly due to differences in the timing and/or diabetes periods that were explored, since T1D involves a progression through several stages^21^. Thus, it would be important to analyse alpha-cell mass and the processes involved in its regulation during key time points of the T1D history in the same diabetic model. These data could provide valuable information to understand the forces involved in the alpha-cell mass dynamics during T1D. The latter is of high actuality in view of recent findings that alpha-to-beta cell transdifferentiation appears to be one of the main mechanisms of beta-cell regeneration^16,22–24^. Additionally, given the involvement of the alpha-cell in the aetiology of T1D and its complications^2,3,9^, a better knowledge about the potential changes in the alpha-cell mass could offer more information about the role of these cells in this disease.

To circumvent caveats associated with either the NOD or STZ mouse model, we opted for the RIP-B7.1 mouse model of experimental autoimmune diabetes (EAD) to study alpha-cell mass in the early-onset and an advanced stage of diabetes. Similar to human T1D^1^, the specific autoimmune destruction of beta-cells in the RIP-B7.1 mouse is mainly driven by CD8^+^ T-cells^25^. In contrast, the autoimmune attack in NOD mice is mainly accomplished by both CD8^+^ and CD4^+^ T-cells^1^, with CD4^+^ T-cell being the predominant subpopulation at diabetes onset^26^. NOD mice may also experience distinctive diseases related to their autoimmune susceptibility that are unlikely to occur in humans^20^, while the EAD-inducible model does not present this limitation^27^. Moreover, T1D models based on STZ-induced beta-cell death do not faithfully recapitulate the autoimmune process against beta-cells characteristic of this pathology and can involve side-effects in multiple organs^20^. Additionally, the diabetic phenotype in the RIP-B7.1 transgenic mice occurs with equal incidence and timing in both sexes in a common mice background, reducing the problem of autoimmune susceptibility genes^27^. Our findings using this stringent EAD model indicate that the alpha-cell population is under an intense attempt of regeneration during the natural course of T1D.

## Results

### Plasma and pancreatic hormone levels are altered one week after diabetes onset

**Figure 1:**
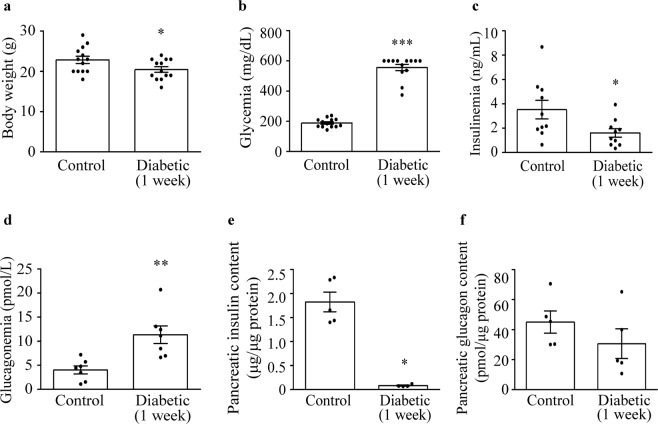
Glycaemia and hormone levels in RIP-B7.1 mice one week after diabetes onset. (**a**) Body weight (n = 13 for each group). (**b**) RIP-B7.1 mice showed hyperglycemia in non-fasting state one week after diabetes onset (n = 13 for each group). (**c**) Diabetic mice presented hypoinsulinemia (n = 10 for each group) and (**d**) hyperglucagonemia (n = 7 for each group) compared with controls. Plasma hormonal measurements were performed in non-fasting state. (**e**) Pancreatic insulin content normalized by total pancreatic protein content (control n = 5, diabetic n = 4). (**f**) Pancreatic glucagon content normalized by total pancreatic protein content (n = 5 for each group). Data presented as mean ± SEM. Unpaired t-test (**a**,**d**,**f**), Mann-Whitney test (**b**,**c**,**e**). Statistical significance is indicated: *p < 0.05; **p < 0.01; ***p < 0.001.

As a result of a beta-cell specific autoimmune attack and destruction, RIP-B7.1 mice develop insulitis, hyperglycaemia and diabetes^25,27,28^. In our study, about 85% of the total immunized animals presented hyperglycaemia after vaccination with preproinsulin (Supplemental Fig. 1). One week after hyperglycaemia onset, diabetic RIP-B7.1 mice featured a lower body weight compared with controls (Fig. 1a). In non-fasting state, diabetic animals were hyperglycaemic (Fig. 1b) and presented an important decrease in plasma insulin levels (Fig. 1c) accompanied with hyperglucagonemia (Fig. 1d), which are characteristic in T1D^5,9^. We next assessed the hormonal content in the whole pancreas. As expected, pancreatic insulin content was severely decreased in diabetic mice (Fig. 1e). In contrast, pancreatic glucagon content did not significantly change (Fig. 1f).

### Pancreatic alpha-cell mass is preserved one week after diabetes onset

**Figure 2:**
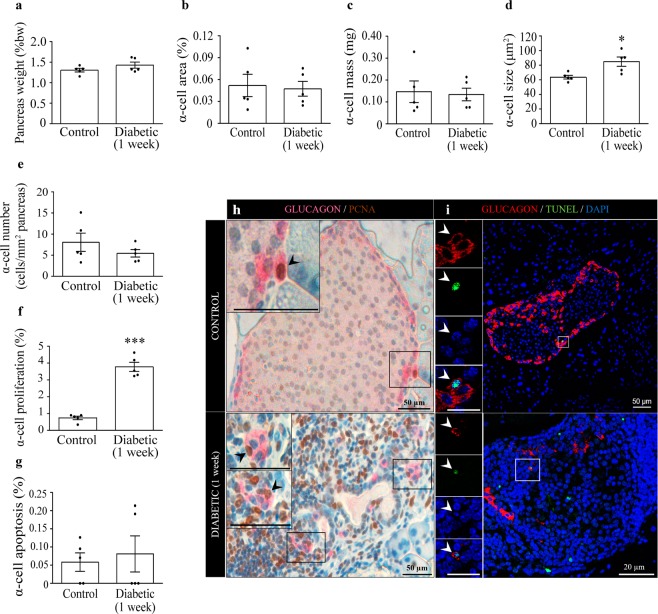
Pancreatic alpha-cell mass, alpha-cell size, proliferation and apoptosis in RIP-B7.1 mice one week after diabetes onset. (**a**) Pancreas weight as percentage of body weight. (**b**) Alpha-cell area expressed as the percentage of the glucagon-positive area over the total pancreatic area. (**c**) Alpha-cell mass. (**d**) Alpha-cell size. (**e**) Alpha-cell number per pancreatic area. (**f**) Alpha-cell proliferation rate: percentage of glucagon and PCNA double-positive cells respect to glucagon-positive cells. (**g**) Apoptosis rate: percentage of glucagon and TUNEL double-positive cells respect to glucagon-positive cells. (**h**) Representative images depicting proliferating alpha-cells. Left panels are enlargements of boxed areas (scale bar: 50 µm). Black arrows indicate PCNA (brown) and glucagon (pink) double-positive cells. (**i**) Representative images of apoptotic alpha-cells in control and diabetic mice. Left panels are enlargements of boxed areas (scale bar: 20 µm). White arrows indicate TUNEL (green) and glucagon (red) double-positive cells. Nuclei were stained with DAPI (blue). N = 5 mice per condition and group in each experiment. Data presented as mean ± SEM. Unpaired t-test (**a**–**f**), Mann-Whitney test (**g**). Statistical significance is indicated: *p < 0.05; ***p < 0.001.

One week after EAD onset, both groups showed similar pancreas weight with respect to the corresponding body weight (Fig. 2a). Islets from diabetic mice exhibited different grades of insulitis (Supplementary Fig. 2a,b). No significant correlation was found between the insulitis grade in each islet and the percentage of islet area occupied by glucagon-positive cells (Pearson’s r = −0.7839, p = 0.11; Supplementary Fig. 2c). We found no differences in alpha-cell area and mass between both groups (Fig. 2b,c). The alpha-cell mass was probably compensated in part by the hypertrophy found in these cells (Fig. 2d), since the relative number of alpha-cells tended to decrease in the diabetic animals (Fig. 2e). Additionally, diabetic mice presented a substantial increase in the number of proliferating alpha-cells compared with controls (Fig. 2f,h), while their apoptotic rate was very low and did not change between both groups (Fig. 2g,i). As expected, the beta cell population was drastically reduced within pancreatic islets of diabetic mice (Supplementary Fig. 3a,c), correlating with the hyperglycaemic state, lower plasma and pancreatic insulin levels and insulitis found in these animals (Fig. 1 and Supplementary Fig. 2). Our results are in agreement with previous reports using this beta-cell specific autoimmune model^25,27–29^.

### Plasma and pancreatic hormone levels are altered after four weeks of EAD

**Figure 3:**
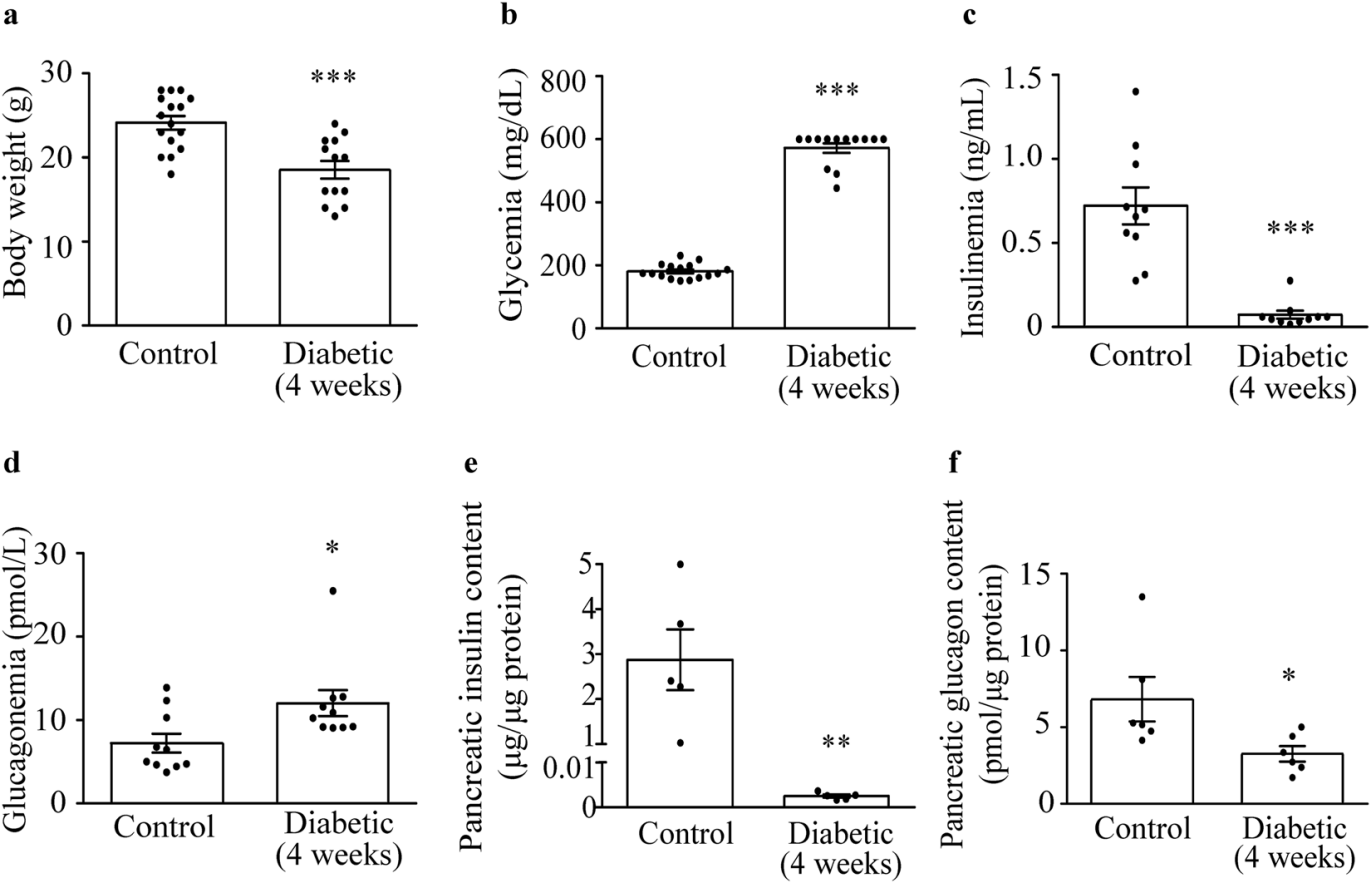
Glycaemia and hormone levels in RIP-B.7.1 mice four weeks after diabetes onset. (**a**) Body weight (control n = 16, diabetic n = 13). (**b**) Glycaemia measured in non-fasting state (control n = 16, diabetic n = 13). Four-week diabetic mice presented hypoinsulinemia (**c**) and hyperglucagonemia (**d**) in non-fasting state (n = 10 for each group). (**e**) Pancreatic insulin (n = 5 for each group) and (**f**) glucagon (n = 6 for each group) contents were decreased in diabetic mice. Both parameters were normalized by total pancreatic protein content. Data presented as mean ± SEM. Unpaired t-test (**a**), Mann-Whitney test (**b**–**f**). Statistical significance is indicated: *p < 0.05; **p < 0.01; ***p < 0.001.

To analyse alpha-cell mass in advanced stages of EAD, we studied RIP-B7.1 animals at four weeks after hyperglycaemia onset. At this stage, diabetic mice also presented a lower body weight compared with controls (Fig. 3a). Similarly to the situation described for the early-onset stage, glycaemic values were highly increased, while both plasma and pancreatic insulin levels were severely reduced (Fig. 3b,c,e). Moreover, hyperglucagonemia was still present (Fig. 3d), while total pancreatic glucagon content exhibited a reduction in diabetic mice (Fig. 3f).

### Pancreatic alpha-cell mass is decreased four weeks after EAD onset

**Figure 4:**
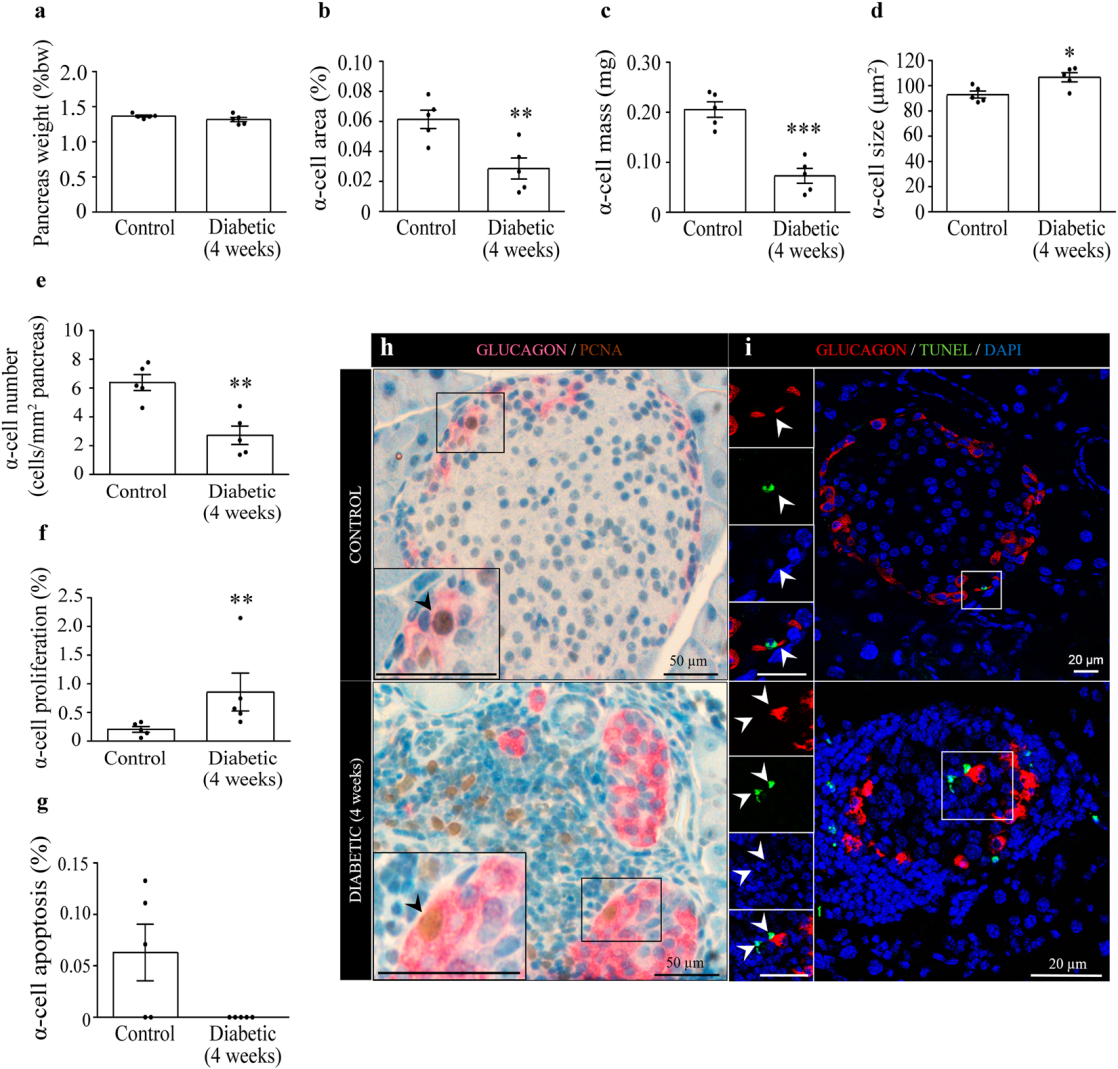
Pancreatic alpha-cell mass, alpha-cell size, proliferation and apoptosis in RIP-B7.1 mice four weeks after diabetes onset. (**a**) Pancreatic weight as percentage of body weight. (**b**) Alpha-cell area expressed as the percentage of the glucagon-positive area over the total pancreatic area. (**c**) Alpha-cell mass. (**d**) Alpha-cell size. (**e**) Alpha-cell number per pancreatic area. (**f**) Proliferation rate: percentage of glucagon and PCNA double-positive cells respect to glucagon-positive cells. (**g**) Apoptosis rate: percentage of glucagon and TUNEL double-positive cells respect to glucagon-positive cells. No TUNEL-positive alpha-cells were found in the pancreas from four-week diabetic mice. (**h**) Representative images from control and diabetic mice depicting proliferating alpha-cells. Left panels are enlargements of boxed areas (scale bar: 50 µm). Black arrows indicate PCNA (brown) and glucagon (pink) double-positive cells. (**i**) Representative images of TUNEL immunofluorescence from control and diabetic mice. Left panels are enlargements of boxed areas (scale bar: 20 µm). White arrows indicate TUNEL (green) and glucagon (red) double-positive cells in control mice, and non-alpha apoptotic cells in diabetic mice. Nuclei were stained with DAPI (blue). N = 5 mice per condition and group in each experiment. Data presented as mean ± SEM. Unpaired t-test (**a**–**e**), Mann-Whitney test (**f**). Statistical significance is indicated: *p < 0.05; **p < 0.01; ***p < 0.001.

We next evaluated the alpha-cell mass in four-week diabetic mice. No changes in the pancreatic weight respect to the body weight were found in the diabetic animals compared with controls (Fig. 4a). Diabetic mice also presented islet infiltration at the advanced phase of the disease (Supplementary Fig. 2a,d), and exhibited a higher insulitis degree compared with the early-onset stage (Supplementary Fig. 2a,b). A significant inverse correlation was found between the insulitis grade in each islet and the percentage of islet area occupied by glucagon-positive cells (Pearson’s r = −0.9745, p < 0.01; Supplementary Fig. 2e). Interestingly, the pancreatic alpha-cell area and mass (Fig. 4b,c) as well as the relative number of alpha-cells (Fig. 4e) were significantly decreased in the pancreas from four-week diabetic mice. Moreover, similarly to the situation found at early-onset, the alpha-cell size and proliferating rate (Fig. 4d,f,h) were elevated at the advanced stage. Alpha-cell apoptosis was very low in controls and no apoptotic events were found in diabetic mice (Fig. 4g,i). At this stage, the beta-cell population was largely reduced in diabetic mice (Supplementary Fig. 3b,c).

### Diabetic mice present glucagon-expressing cells in the ductal epithelium

**Figure 5:**
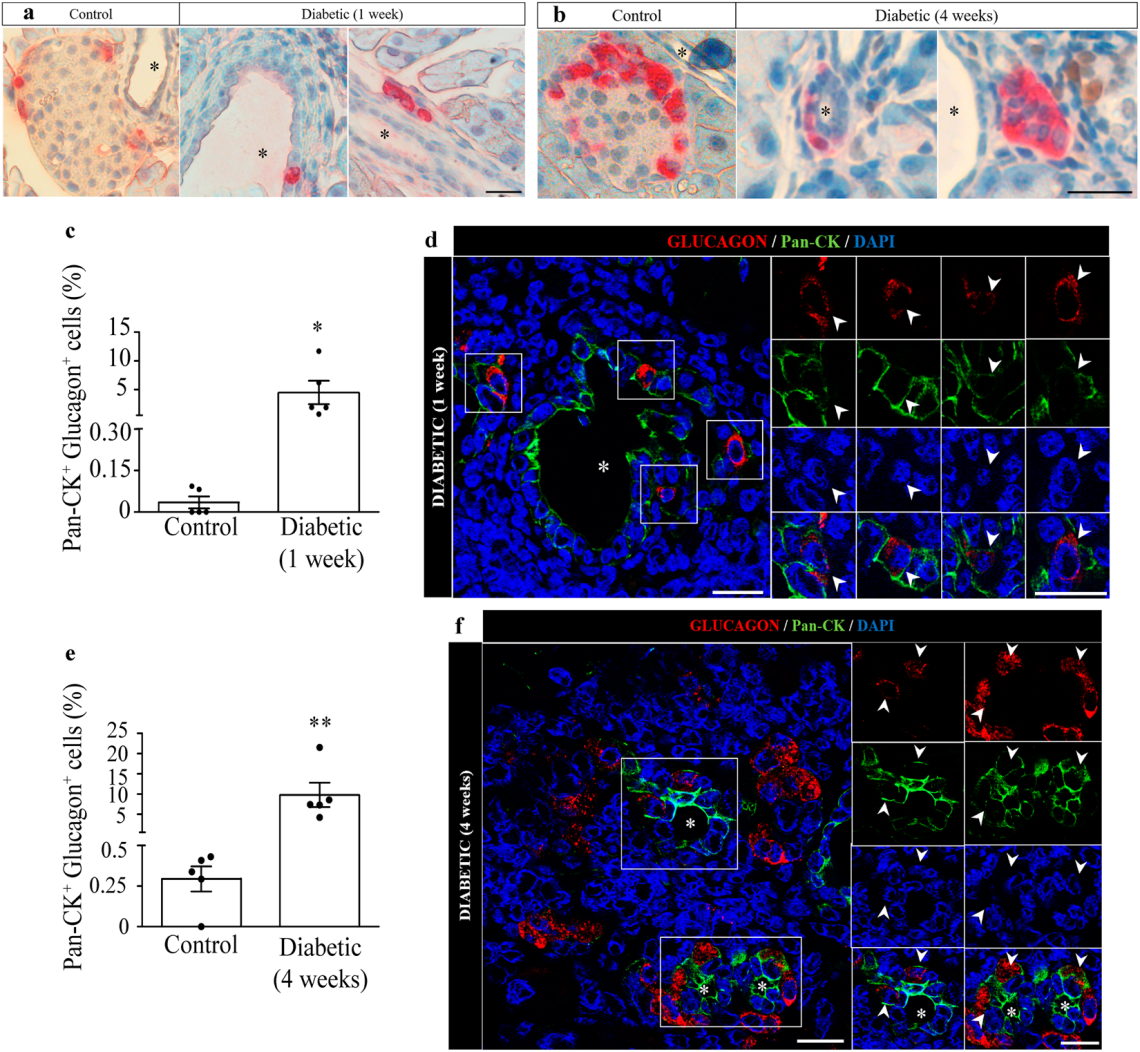
Quantification of pancreatic cells expressing both glucagon and the ductal epithelium marker pan-CK in one-week and four-week diabetic RIP-B7.1 mice. Representative images of glucagon-positive cells (pink) within or near the ductal epithelium in two different one-week (**a**) and four-weeks (**b**) diabetic mice and their respective controls. Scale bar: 50 µm. (**c**) Percentage of cells positive for glucagon and the ductal marker pan-CK respect to total alpha-cells in the pancreas from one-week diabetic mice. (**e**) Percentage of cells positive for glucagon and the ductal marker pan-CK respect to total alpha-cells in the pancreas from four-week diabetic mice. (**d**,**f**) Representative images of double-positive cells expressing pan-CK (green) and glucagon (red) in one-week (**d**) and four-week (**f**) diabetic mice. Nuclei were stained with DAPI (blue). Right panels show enlargements of boxed areas. White arrows indicate double-positive cells. Pancreatic ducts are indicated (*). N = 5 mice per group. Scale bar: 20 µm. Control representative images are shown in Supplemental Fig. 4. Data presented as mean ± SEM. Mann-Whitney test. Statistical significance is indicated: **p < 0.01.

In both stages, one and four weeks after EAD onset, we observed the presence of glucagon-positive cells near or within the pancreatic ducts from diabetic mice, while these cells were infrequent in control animals (Fig. 5a,b). In light of this observation, and since neogenesis from pancreatic ductal epithelium could be a potential contributor to the endocrine cell mass^30–32^, we assessed the presence of alpha-cells that could derive from ductal epithelia by analysis of those cells that were positive for both cytokeratin (pan-CK) and glucagon. Interestingly, there was an increased percentage of cells positive for both markers in one-week diabetic animals (Fig. 5c,d; Supplementary Fig. 4a), suggesting a process of cell renewal from the ducts. The presence of these double-positive cells persisted four weeks after diabetes onset (Fig. 5e,f; Supplementary Fig. 4b).

### Bi-hormonal cells expressing both insulin and glucagon are increased in the pancreas of diabetic mice

**Figure 6:**
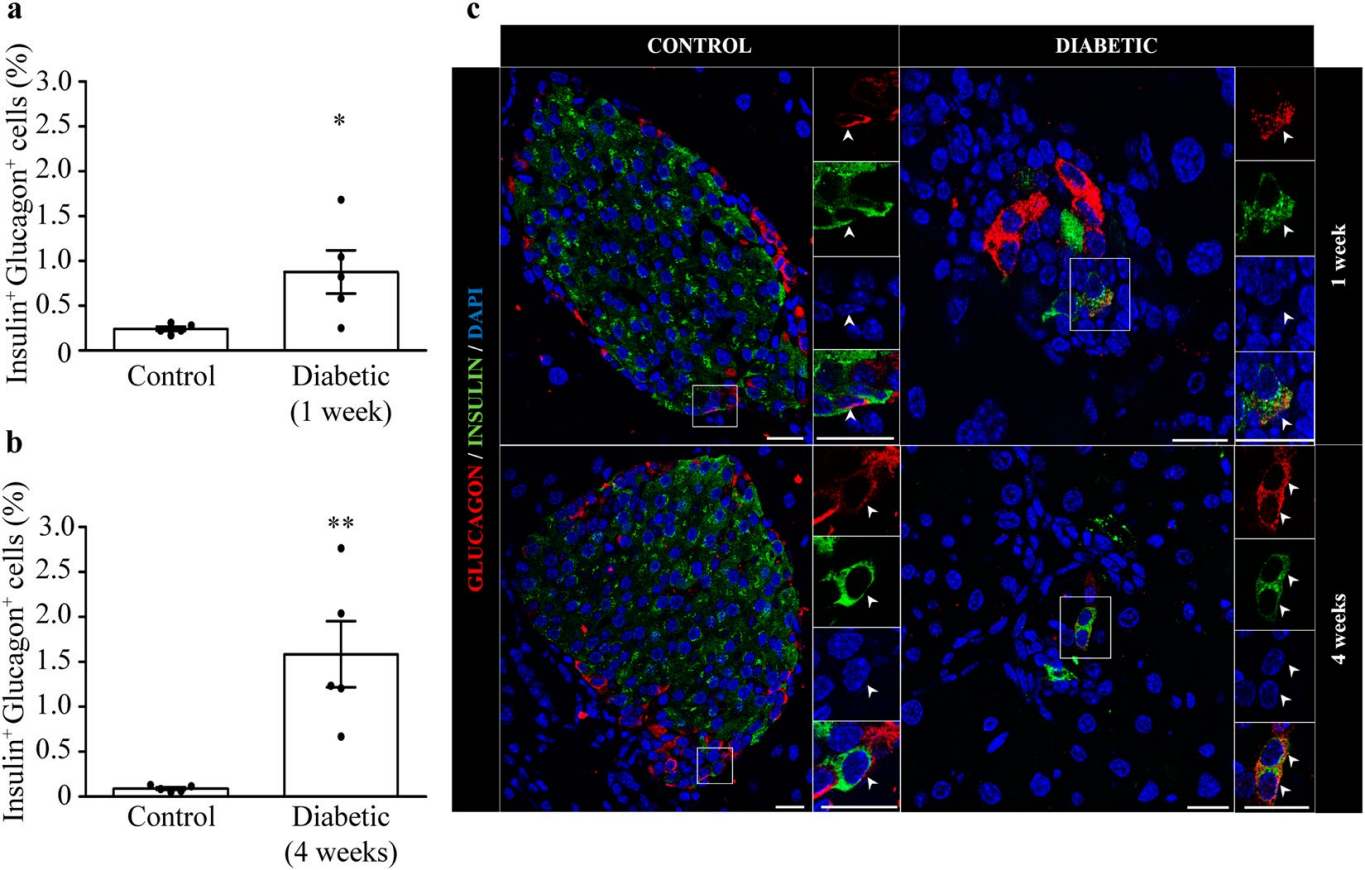
Increase of bi-hormonal cells expressing both insulin and glucagon in the pancreas of one-week and four-week diabetic mice. Percentage of bi-hormonal cells positive for insulin and glucagon respect to glucagon-positive cells in control and diabetic mice one week (**a**) and four weeks (**b**) after diabetes onset. (**c**) Representative images showing double-positive cells for glucagon (red) and insulin (green) in control and diabetic mice. Right panels are enlargements of boxed areas with white arrows indicating double-positive cells. Nuclei were stained with DAPI (blue). Scale bar: 20 µm. N = 5 mice per group. Data presented as mean ± SEM. Mann-Whitney test. Statistical significance is indicated: *p < 0.05; **p < 0.01.

**Figure 7:**
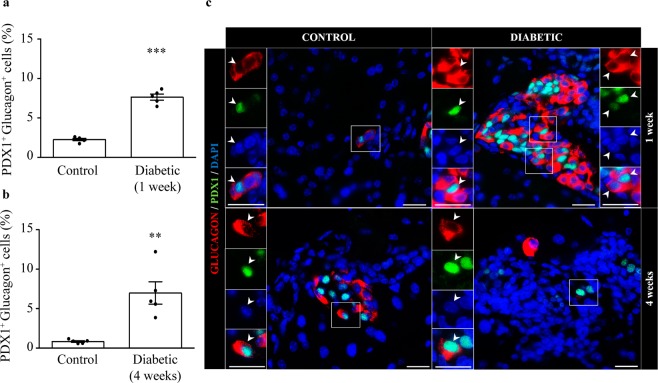
Increase of cells expressing both PDX1 and glucagon in the pancreas of one-week and four-week diabetic mice. Percentage of cells positive for PDX1 and glucagon respect to total glucagon-positive cells in control and diabetic mice one week (**a**) and four weeks (**b**) after diabetes onset. (**c**) Representative images showing double-positive cells for PDX1 (green) and glucagon (red) in control and diabetic mice. Left panels are enlargements of boxed areas with white arrows indicating double-positive cells. Nuclei were stained with DAPI (blue). Scale bar: 20 µm. N = 5 mice per group. Data presented as mean ± SEM. Unpaired t-test (**a**), Mann-Whitney test (**b**). Statistical significance is indicated: **p < 0.01; ***p < 0.001.

We next evaluated whether alpha-to-beta cell transdifferentiation could be involved in the regulation of the alpha-cell mass during EAD. For this purpose, we analysed the presence of islet cells expressing both insulin and glucagon. We found a significant increase in the percentage of bi-hormonal cells in both one-week and four-week diabetic mice compared with controls (Fig. 6), in which the presence of double-positive cells was negligible. To further confirm these findings, we evaluated the presence of cells positive for glucagon and PDX1, a transcription factor specifically expressed in postnatal immature and mature beta-cells^33^. The percentage of cells positive for glucagon and PDX1 was increased in diabetic animals at both stages (Fig. 7).

## Discussion

Research in T1D has focused mainly on the pancreatic beta-cell mass and its regeneration^1^. However, despite the involvement of the pancreatic alpha-cell in the aetiology of this disease and its complications^2,3,9^, little is known about this islet cell population in T1D. In the present study, we have analysed the alpha-cell mass in an EAD-inducible model, the RIP-B7.1 mouse. As mentioned earlier, the RIP-B7.1 mouse appears to possess most of the human attributes to study alpha-cell mass in T1D.

Human T1D is associated with hyperglucagonemia, which can exacerbate the hyperglycaemic state^9^. Unfortunately, this condition is not faithfully recapitulated in all mouse models. For instance, NOD mice, the most used EAD model, do not consistently exhibit hyperglucagonemia with diabetes evolution^18^, while this situation is present in pre-diabetic states^7^. In contrast, STZ-induced diabetic mice show a significant increment in plasma glucagon^6^. Herein, we demonstrate that RIP-B7.1 mice develop hyperglucagonemia during the course of EAD mimicking the human phenotype. Given that a hypoinsulinemic intra-islet environment can favour glucagon hypersecretion^2,3,34^, the high glucagon levels of diabetic RIP-B7.1 mice were most probably related with the low plasma and pancreatic insulin levels found in these animals.

Analysis of the alpha-cell mass in different T1D models has resulted in diverse outcomes. Plesner and colleagues reported an increase of the alpha-cell mass in the STZ single-dose model^18^. In contrast, the alpha-cell mass was unaltered with disease progression in either NOD mice^18^ or in the STZ multiple low-dose model^6,19^. Given that beta-cell death in NOD mice and in the STZ multiple low-dose model results in islet inflammation^20^, while this process is absent in the single STZ dose model^20^, it is possible that pancreatic insulitis may restrict alpha-cell expansion. Consistent with this premise, an inverse correlation between the insulitis grade and the proportion of alpha-cells was observed in islets of four-week diabetic RIP-B7.1 mice. In this context, both alpha-cell mass and pancreatic glucagon content were found to be preserved in our one-week diabetic mice, as has been reported in T1D mouse models with islet infiltration^6,18,19^ and recent-onset T1D patients^12^. In contrast, at four weeks after diabetes onset, both parameters were diminished. This decrease is consistent with studies conducted in patients primarily with long-term T1D^13,14^, suggesting that the alpha-cell pool is gradually depleted with disease progression. In any case, further studies are necessary to stablish whether the inflammatory process as well as other factors such as the hyperglycaemic environment can modulate the pancreatic alpha-cell mass during T1D.

Interestingly, alpha-cell size, proliferation and neogenesis were increased at both stages in diabetic RIP-B7.1 mice, whereas no significant changes in alpha-cell apoptosis were observed, suggesting an active cellular renewal during the course of EAD. In agreement with our findings, increased human alpha-cell proliferation has been reported at the recent onset of T1D^17^. Conversely, reduced values have been obtained in young adults and children with T1D^15^. In the case of animal models, different results have been documented. While studies with mice treated with multiple STZ low-dose showed either an increased^8^ or similar^19^ alpha-cell proliferation rate, this parameter was found to be reduced in diabetic NOD mice^18^. A low alpha-cell apoptotic rate in the diabetic animals was not unexpected since rodent and human alpha-cells display a high apoptosis resistance in diabetic environments due to a specific set of proteins that allow their survival^35,36^. This indicates that apoptosis may not play a significant role in the regulation of alpha-cell mass during EAD. In any case, we cannot discard that previous apoptotic events might contribute to the decreased alpha-cell mass observed in four-week diabetic animals. Consistent with findings in NOD mice^30^, we detected a group of glucagon-positive cells expressing a ductal epithelium marker, which suggests the presence of neogenic processes from the ducts in T1D. Although ductal neogenesis probably does not contribute to the endocrine cell mass in adult mice under physiological situations^31^, this process can represent a potential source of new beta-cells under experimental and pathological conditions^32,37^. Proliferation and neogenesis try to compensate for beta-cell loss during T1D progression in an attempt to regenerate the beta-cell population^37^. Our results suggest that both processes perform a similar role to maintain the alpha-cell population in RIP-B7.1 diabetic mice.

The lack of apoptosis combined with stimulated cell proliferation and neogenesis would argue in favour of increased alpha-cell mass. Yet we observed a decrease in alpha-cell mass during the course of diabetes, suggesting that other cellular processes were contributing to the overall alpha-cell population. In this context, we found a higher percentage of bi-hormonal cells expressing insulin and glucagon in both one- and four-week diabetic RIP-B7.1 mice, substantiating previous reports showing that alpha-to-beta cell transdifferentiation could be an important spontaneous route of beta-cell regeneration in several EAD mouse models^22,24,28,38,39^ and in human T1D subjects^16,40^. Several works have shown that alpha-to-beta cell transdifferentiation takes place following a bi-hormonal intermediary expressing both insulin and glucagon^16,22^. In contrast, beta-cells do not seem to spontaneously transdifferentiate to glucagon-expressing cells after near total alpha-cell loss^41^, indicating that alpha-to-beta cell reprogramming may be a prevalent process in diabetes. We also detected a higher percentage of alpha-cells labelled for PDX1, which is specifically expressed in immature and mature postnatal beta-cells^33^. The presence of alpha-cells expressing this transcription factor has also been reported in beta-cell loss models^16,22,38^ and in T1D patients^23,42^. Additionally, forced expression of PDX1 along with MAFA induces alpha-to-beta transdifferentiation in NOD mice and STZ-treated human islets^43,44^. Thus, the occurrence of glucagon-positive cells expressing insulin and/or PDX1 seems to be more related with a process of alpha-to-beta reprogramming^16,22,38,39,45^ rather than alpha-cell dedifferentiation to an early endocrine progenitor^33^. Additionally, no bi-hormonal cells have been identified in mouse models of beta-cell dedifferentiation^46^. Taken together, our results suggest that alpha-to-beta cell transdifferentiation may contribute long-term to the decrease of the alpha-cell mass in diabetic RIP-B7.1 mice. These findings also indicate that the increased alpha-cell turnover observed in EAD could be associated with the maintenance of an alpha-cell pool to replenish beta-cells, as previously reported for the inactivation of *Arx* in alpha-cells, which induces both alpha-to-beta transdifferentiation and, subsequently, alpha-cell ductal neogenesis^47^. Alternatively, the increased regeneration observed in our model might be a compensatory response to the loss of functional alpha-cells in order to maintain the alpha-cell population and its related functions. Newly formed beta-cells are more vulnerable to cytokine-induced cell death^48^. Thus, this could constrain the alpha-cell capacity to repopulate beta-cells at the expense of its own mass when facing increasing cytokine levels.

While the presence of glucagon-positive cells expressing insulin and/or PDX1 have been documented in several EAD models^16,22,24,38,39^ and in T1D donors^16,40,42^, other studies have not found these bi-hormonal cells in T1D patients^49^. Thus, it is possible that the occurrence of transdifferentiation events may depend on some variables such as subject age, diabetes duration, glycaemic and immune conditions, as well as stress or injury level, as has been suggested from diabetes mouse models^22,24,50^ and human T1D studies^23,49^. It has been also shown that the frequency of bi-hormonal cells could change when considering the time after beta-cell loss^50^. In this regard, the proportion of insulin/glucagon double-positive cells has not been found to be increased in RIP-B7.1 mice when analysed at eight weeks after immunization^28^. However, it is important to mention that the conditions of EAD and analysis were different from those used in our present study. The former report was focused on the immune process and used mice at four and eight weeks after immunization, which implied about 50% and 80% of diabetes incidence, respectively^28^. In contrast, all mice employed in the present study were diabetic, taking into account the moment of diabetes onset: one week and four weeks after the development of hyperglycaemias. In our case, the one-week diabetic group involved a period after immunization that ranged from 3 to ~7 weeks, while the four-week group implicated a period of about 7 to 15 weeks after immunization. Thus, it is difficult to compare both studies given that animals were at different stages of immunization and diabetes, and it may lead to different outcomes, as mentioned above^22–24,49,50^. The findings of the current research, in which all animals were diabetic, are consistent with different studies in T1D mouse models and T1D human pancreata showing an increase in bi-hormonal cells^22–24,40^. Four weeks after immunization, alpha-cell mass was also found to be reduced in RIP-B7.1 mice^28^. Given that T-cell infiltration in islets from RIP-B7.1 mice is already detected two weeks after immunization^25^, it is possible that the autoimmune attack could modulate alpha-cell mass before diabetes onset^21^. Therefore, experimental conditions, diabetes stages and immunization times, among other factors, appear to be important to analyse alpha-cell mass during EAD, as suggested in different studies^22–24,49,50^. Overall, all these findings indicate that the alpha-cell mass and the processes involved in its regulation are dynamic and present high plasticity during the course of EAD.

In summary, we report here that the alpha-cell population is preserved at the early-onset of autoimmune diabetes, in contrast to the known situation of the beta-cells^1^. Pancreatic alpha-cell mass, however, undergoes a decrease at advanced diabetes. In both stages, several processes involved in the control of islet cell mass such as cell size, proliferation and ductal neogenesis were up-regulated, while apoptosis was almost negligible. Additionally, an increased rate of bi-hormonal cells and glucagon/PDX1-positive cells was detected, suggesting a process of alpha-to-beta transdifferentiation. Thus, our findings indicate that, in autoimmune diabetes, alpha-cells are facing an intense regenerative state in an effort to maintain alpha-cell mass and/or to sustain a cell pool directed to regenerate beta-cells. These results also support that pancreatic alpha-cells present a significant capability to adapt to EAD, and that this plasticity may be potentially used as an approach to beta-cell regeneration in T1D.

## Methods

### Mouse model and EAD induction

The murine model of experimental autoimmune diabetes RIP-B7.1 (H-2^b^) C57BL/6 J express the immune system stimulatory molecule B7.1 (CD80) under the rat insulin promoter (RIP) in pancreatic beta-cells, increasing the beta-cell susceptibility to autoimmune destruction^25,27^. EAD was induced by DNA immunization as previously described^25^. For experimental studies, male and female heterozygous F1 animals (C57BL/6J × RIP-B7.1) were used at 12.5 ± 0.42 weeks for the early-onset study and at 15.8 ± 0.46 weeks for the advanced stage study. DNA immunization was performed as previously described^27^. Briefly, 50 µg of plasmid DNA (dissolved in 50 µl PBS) expressing murine preproinsulin II (Plasmid Factory, Germany) was injected into each tibialis anterior muscle. Age and sex-matched heterozygous RIP-B7.1 control mice were injected with only PBS. Blood samples were collected from tail vein and glycemia was monitored every two days using an automatic glucometer (Accu-Chek Compact plus, Roche). Mice were considered diabetic when two consecutive blood glucose measurements in non-fasting state exceeded 250 mg/dl^27^. To study the alterations in the early-onset of EAD, experiments were performed one week after diabetes initiation in mice originally housed in CABIMER animal facilities, which were sent to the Miguel Hernández University immediately after their immunization. For the study of mice with established diabetes, experiments were carried out four weeks after diabetes onset in mice housed in the Miguel Hernández University animal facilities. In the present study, about 85% of the total immunized animals presented hyperglycaemia and developed diabetes after the vaccination with preproinsulin. Only those immunized mice that developed hyperglycaemia were studied in the corresponding diabetic group. All studies were approved by the Animal Ethics Committee of the Miguel Hernández University according to national regulations and approved guidelines.

### Plasma and total pancreatic hormone measurements

Plasma glucose, insulin, and glucagon levels were measured in non-fasting state^51^. For hormonal measurements, blood was collected by decapitation after CO_2_ anaesthesia with a K2 EDTA coated Microvette tube (Sarstedt) and in the presence of 500 KIU/ml aprotinin (Sigma-Aldrich). Then, blood was centrifuged at 200 g for 20 minutes at 4 °C and plasma was collected and stored at −80 °C. To measure pancreatic glucagon and insulin content, pancreata were dissected and placed in a tube containing 5 ml of acid-ethanol buffer (75% EtOH, 1.5% HCl). After overnight incubation at −20 °C, samples were homogenised and reincubated overnight again at −20 °C. Afterwards, extracts were centrifuged at 400 g for 15 minutes at 4 °C and supernatants were neutralized with 1 M Tris (pH 7.4) before ELISA measurements. Results were normalized by the total protein pancreatic content measured by the Bradford assay. Glucagon and insulin levels were assessed by ELISA (Mercodia, #10-1271-01; Crystal Chem, #90080; respectively) following commercial instructions.

### Immunohistochemistry and proliferation analysis

Pancreata were dissected, cleared from fat and lymph nodes and fixed with 4% paraformaldehyde in PBS for 22 hours at 4 °C. Then, pancreata were weighted, embedded in paraffin and sectioned. For immunohistochemical studies, two to three pancreatic sections (5 µm) were cut 200 µm apart^51^. The whole pancreatic section was examined. To study alpha-cell mass and proliferation, the kit EnVision G/2 Doublestain System (DAB+/Permanent Red) was used following commercial instructions (DAKO K5361, Agilent Technologies). Antigen retrieval was performed by heating the samples with a microwave in citrate buffer (12 mM, pH 6.0). The slides were blocked by incubation for 2 hours in 3% BSA in PBS. For alpha-cell detection, rabbit anti-glucagon antibody (1:100, PS087, Monosan) was used. Proliferation was analysed using the proliferating cell nuclear antigen (mouse anti-PCNA, 1:4000, #2586, Cell Signalling) labelling technique^51^. Both antibodies were incubated overnight at 4 °C. Samples were counterstained with haematoxylin-eosin for nuclei counting and mounted with Faramount aqueous mounting medium (DAKO S3025, Agilent Technologies). Stained slides were imaged at 4x for quantification of total pancreatic area, and at 20x for pancreatic islets and glucagon-stained area. A Kappa ACC1 camera attached to a Nikon Eclipse TE200 Microscope was used for the early-onset study, while an Olympus IX71S8F-2 Microscope with an Image Development Systems camera was used for the long-term study. Total pancreatic and glucagon-positive area were measured in each slide using the Metamorph Analysis Software (Nashville, TN, USA) according to previous reports^51,52^. Pancreatic alpha-cell area was calculated by measuring the total glucagon-stained area normalized by the total pancreatic area. Alpha-cell size was evaluated by dividing the alpha-cell area by the total number of alpha-cell nuclei. Alpha-cell mass was then calculated multiplying the alpha-cell area by the pancreas weight. Proliferation rate was expressed as percentage of alpha-cells with PCNA-positive nuclei respect to the total alpha-cells.

### Immunofluorescence analysis

Pancreata were collected, fixed and processed as described above. Alpha-cell apoptotic rate was analyzed by TUNEL as previously described^51,52^, using the manufacturer’s protocol for difficult tissue (*In Situ* Cell Death Detection Kit, Roche). After dehydration, antigen retrieval was performed by heating the samples with a microwave in citrate buffer (12 mM, pH 6.0) for 20 minutes followed by a treatment during 20 minutes with Tris-HCl buffer (0.1 M, pH 7.5, 3% BSA, 20% FBS). Then, slides were incubated with TUNEL reaction mix for 1 hour at 37 °C. Afterwards, slides were blocked in 3% BSA for 2 hours before incubation with a rabbit anti-glucagon antibody (1:100, PS087, Monosan) at 4 °C overnight^51^. To assess alpha-cell neogenesis from pancreatic duct epithelium, antigen retrieval was also performed by heating the samples with a microwave in citrate buffer (12 mM, pH 6.0) followed by incubation with trypsin (0.5 mg/ml) at 37 °C for 30 minutes. Then, samples were blocked by incubation in 3% BSA for 2 hours. Slides were incubated overnight at 4 °C with rabbit anti-glucagon (1:100, PS087, Monosan) and mouse anti-pan-Cytokeratin (1:300, pan-CK, sc-8018, Santa Cruz Biotechnology) antibodies. The presence of bi-hormonal cells expressing both insulin and glucagon was studied by the incubating slides with antibodies against insulin (1:100, rabbit anti-insulin, #C1212, Santa Cruz Biotechnology) and glucagon (1:100, mouse anti-glucagon, C2654, Sigma), after antigen retrieval with citrate buffer (12 mM, pH 6.0) and blocking for 2 hours with 3% BSA. Likewise, PDX1 expression in glucagon-positive cells was assessed using guinea pig anti-PDX1 antibodies (1:200, ab47308, abcam).

For fluorescence detection, all samples were incubated with the corresponding secondary antibodies, Alexa Fluor 546 or 488 (1:500, Life Technologies), for 2 hours at room temperature. Total nuclei were stained with Hoechst 33342 (1:1000, Invitrogen) and sections were mounted using ProLong Gold Antifade Reagent (P36930, Invitrogen). Images were acquired using a Zeiss Axio Observer Z1 microscope (Carl Zeiss) equipped with an ApoTome system, which allowed obtaining optical sections (0.7 μm) with an objective EC Plan-NEOFLUAR 40x. Images were analysed using Metamorph Analysis Software (Nashville, TN, USA). Individual glucagon-positive cells were assessed manually for double labelling following two criteria: single nucleus surrounded by a glucagon-positive cytoplasm and matching staining of nucleus or cytoplasm with the antibody of interest. Negative controls were obtained using samples in which the complete protocol was performed except for the incubation with the primary antibodies. A positive control was also used for the TUNEL analysis, as indicated in the manufacturer’s instructions.

### Insulitis and glucagon score

As previously reported^18,39^, insulitis and glucagon scoring were performed according to the percentage of infiltrated/glucagon islet area: grade 0, 0%; grade 1, <10%; 10% < grade 2 >55%; 55% < grade 3 >75%; grade 4 >75%. Note that insulitis grade 0 involves pre-inflammation islets as well as post-inflammation islets where infiltration has dispersed. Insulitis and glucagon were scored in the same pancreatic sections employed for alpha-cell mass and proliferation measurements, and thus, PCNA-positive cells (labelled in brown) are also observed in the Supplementary Fig. 2. As in similar studies^18,39^, immune cells in infiltrated islets were easily visualized with the haematoxylin staining by their characteristic dense nuclei and small size.

### Data representation and analysis

Statistical analysis was performed with GraphPad Software v5. After testing for normality, we performed Student’s t-test or the nonparametric test Mann-Whitney when variables were not normally distributed. Two-way ANOVA with Bonferroni correction was employed when required. Data are presented as mean ± SEM and statistical significance was set at p < 0.05 for all analyses.

## Supplementary information

Supplementary material

## Data Availability

The datasets generated during and/or analysed during the current study are available from the corresponding author on reasonable request.
