# Supplementary material for: Pancreatic alpha-cell mass in the early-onset and advanced stage of a mouse model of experimental autoimmune diabetes
Source: Sci Rep. 2019 Jul 2;9:9515. doi: 10.1038/s41598-019-45853-1 (PMC6606577; doi:10.1038/s41598-019-45853-1)
Supplement: Supplementary file 1 — Supplementary material [file 41598_2019_45853_MOESM1_ESM.pdf]

Pancreatic alpha-cell mass in the early-onset and advanced stage of a mouse model of experimental autoimmune diabetes.

Eva Bru-Tari[1], Nadia Cobo-Vuilleumier[2], Paloma Alonso-Magdalena[1], Reinaldo S. Dos Santos[1], Laura Marroqui[1], Angel Nadal[1], Benoit R. Gauthier[2], Ivan Quesada[1*].

[1]Instituto de Investigación, Desarrollo e Innovación en Biotecnología Sanitaria de Elche (IDiBE), IBMC and Biomedical Research Center in Diabetes and Associated Metabolic Disorders (CIBERDEM), Universidad Miguel Hernández, Elche, Spain.

[2]Department of Cell Regeneration and Advanced Therapies, Andalusian Center for Molecular Biology and Regenerative Medicine-CABIMER, Junta de Andalucia-University of Pablo de Olavide-University of Seville-CSIC, Seville, Spain.

*Corresponding author:

I. Quesada. Instituto de Investigación, Desarrollo e Innovación en Biotecnología Sanitaria de Elche (IDiBE), Universidad Miguel Hernández de Elche, Avenida de la Universidad s/n, 03202 Elche, Spain. Email: ivanq@umh.es

SUPPLEMENTAL FIGURE 1

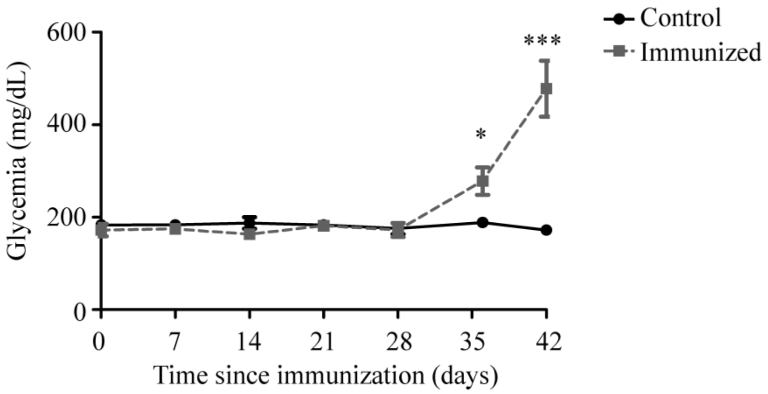

Supplemental figure 1. Plasma glucose changes in RIP-B7.1 mice after immunization. Time course of non-fasting glycemic values of a group of animals that developed diabetes at the same temporal range after preproinsulin DNA vaccine administration at day 0 (n=5 mice per condition). Data presented as mean ± SEM. Two-way ANOVA. Statistical significance is indicated: *, $p<0.05$; ***, $p<0.001$.

SUPPLEMENTAL FIGURE 2

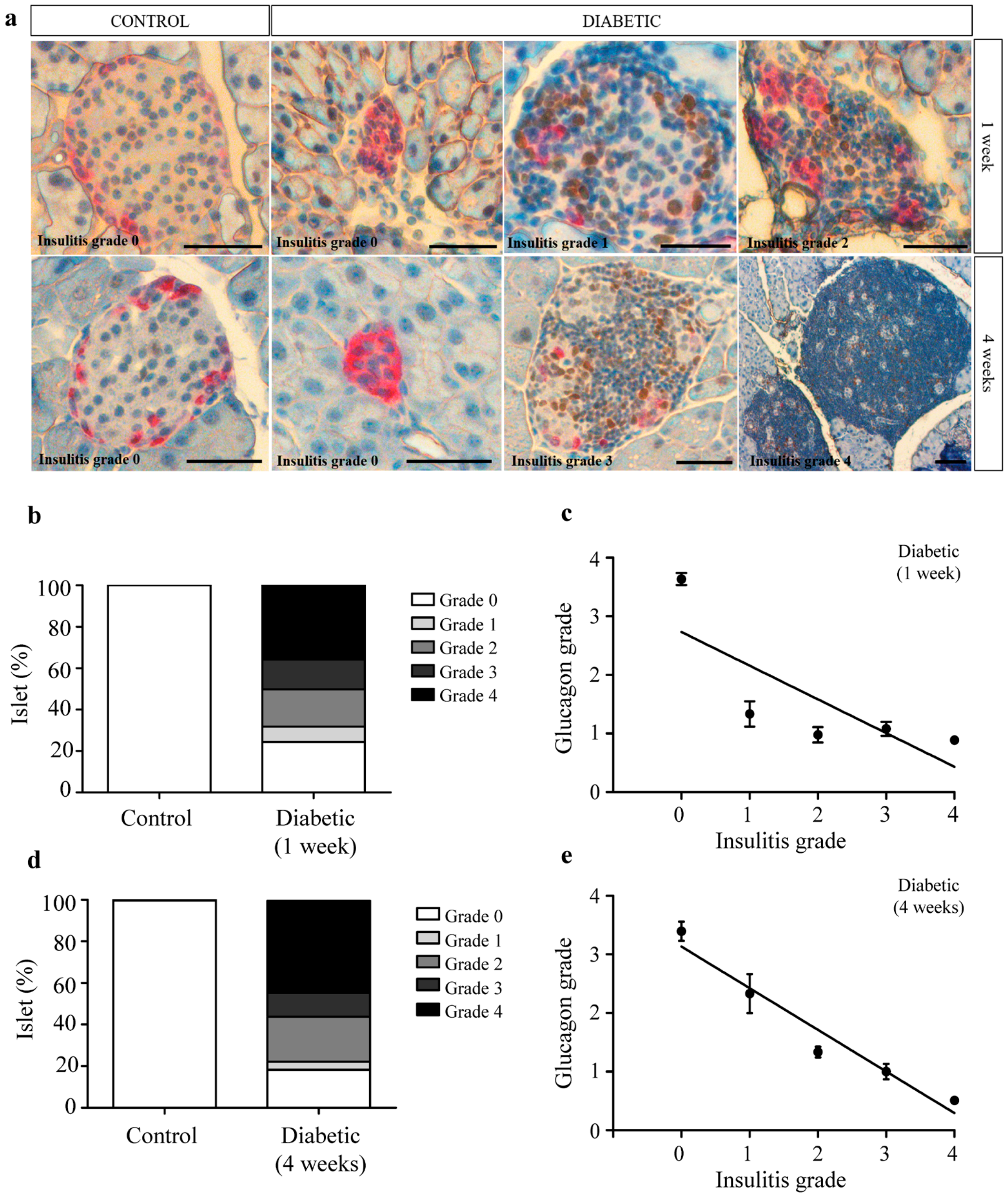

Supplemental figure 2. Insulitis and glucagon grades in pancreatic islets from diabetic mice and controls. (**a**) Representative images of pancreatic islets from controls and one-week or four-week diabetic animals are shown. Images illustrate different degrees of insulitis. Glucagon staining is labeled in pink, while nuclei were counterstained with hematoxylin-eosin. Scale bar: 50 µm. (**b**) Insulitis scoring of islets from one-week diabetic mice and controls represented as percentage of total islet number (n=288 islets from 5 control mice; n=255 islets from 5 diabetic mice). (**c**) Trend

to negative correlation between the islet percentage stained for glucagon (glucagon grade) and the insulitis grade in one-week diabetic mice (Pearson r = -0.7839, p=0.11). (**d**) Insulitis scoring of islets from four-week diabetic mice and controls (n=352 islets from 5 control mice; n=234 islets from 5 diabetic mice). (**e**) Negative correlation between the islet percentage stained for glucagon (glucagon grade) and the insulitis grade in four-week diabetic mice (Pearson r = -0.9745, p<0.01).

SUPPLEMENTAL FIGURE 3

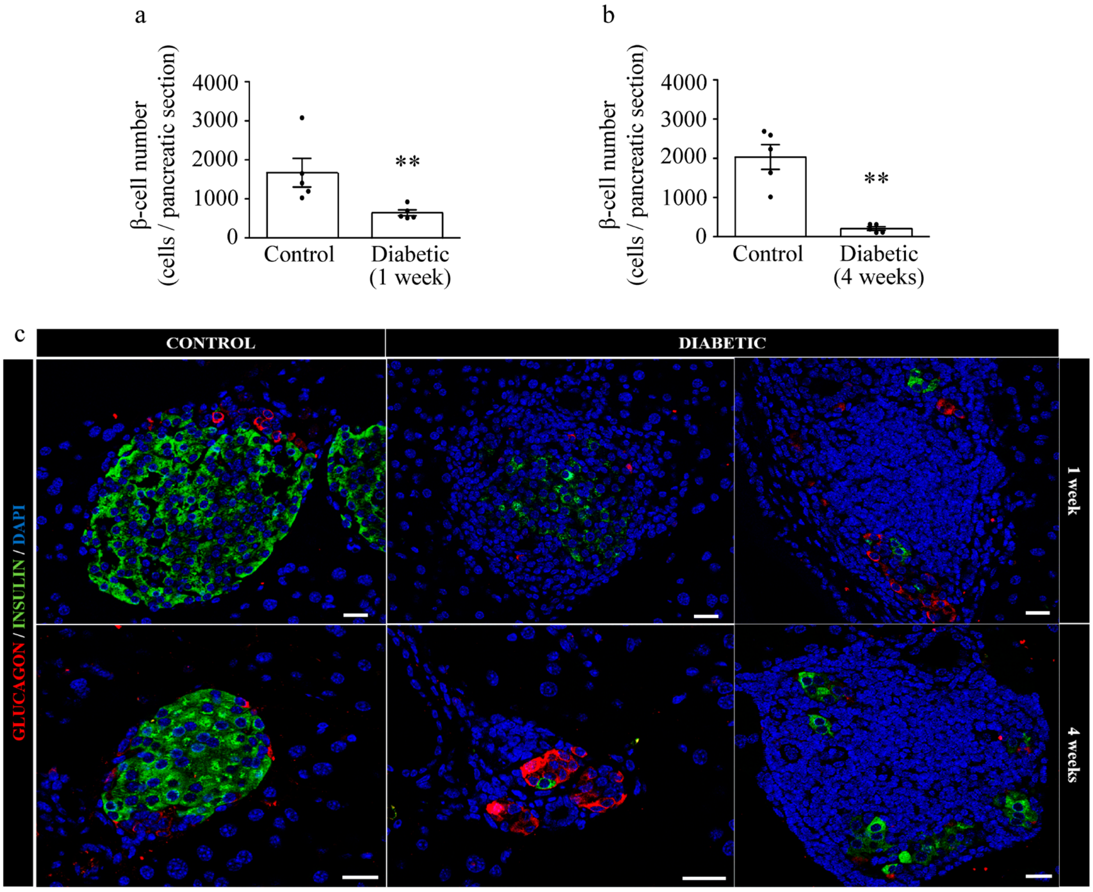

Supplemental figure 3. Beta-cell number in the pancreas from control and diabetic mice. Beta-cell number per pancreatic section in one-week **(a)** and four-weeks **(b)** diabetic mice. All insulin-positive cells from two pancreatic sections were counted for each mouse (n=5 mice per group). Each pancreatic section contained the whole tissue area. (**c**) Representative images of pancreatic islets from two different diabetic mice in both EAD stages, and their corresponding controls. Insulin is labelled in green, glucagon in red and total nuclei in blue. Data presented as mean ± SEM. Unpaired t-test. Statistical significance is indicated: **, $p<0.01$.

SUPPLEMENTAL FIGURE 4

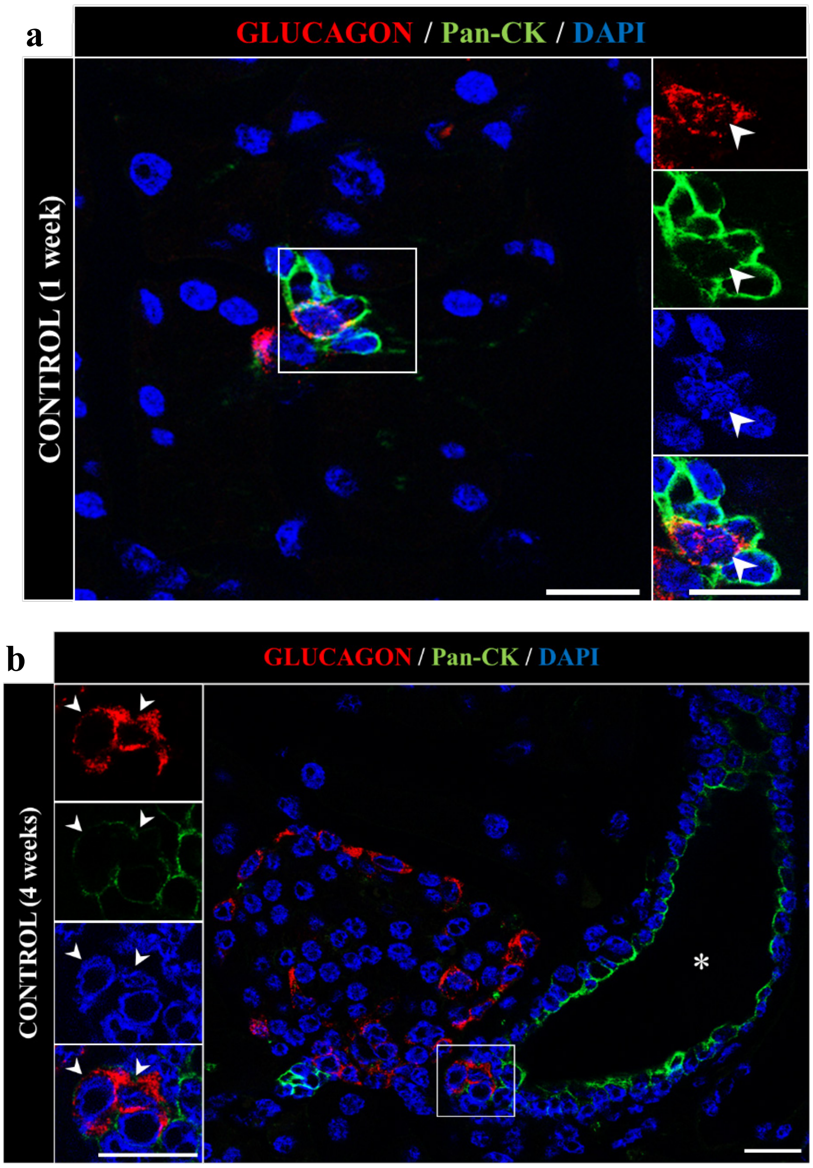

Supplemental figure 4. Representative images of control mice from figure 5. Representative images of double-positive cells expressing pan-CK (green) and glucagon (red) in the pancreas of control mice. (**a**) representative control image of the one-week diabetic group. (**b**) representative control image of the four-weeks diabetic group. Nuclei were stained with DAPI (blue). Right and left panels show enlargements of boxed areas. White arrows indicate double-positive cells. Pancreatic ducts are indicated (*). Scale bar: 20 μm
